# Multidisciplinary management of severe open pelvic fracture with multiple organ injuries: A case report

**DOI:** 10.1097/MD.0000000000043551

**Published:** 2025-08-01

**Authors:** Haixiang Ding, Wenwen Wang, Mingyang Yuan, Junjan Tang, Jianhong Zhou, Jingshi Pan

**Affiliations:** a Department of Trauma Surgery, Affiliated Hospital of Jiangnan University, Wuxi, Jiangsu, China; b Department of Pharmacy, Wuxi Health Vocational College, Wuxi, Jiangsu, China; c Department of Vascular Surgery, Affiliated Hospital of Jiangnan University, Wuxi, Jiangsu, China.

**Keywords:** case report, damage control surgery, multidisciplinary management, open pelvic fractures, staged surgical approach

## Abstract

**Rationale:**

Open pelvic fractures represent severe traumatic injuries with mortality rates approaching 50%. Complex cases involving multiple organ injuries and massive hemorrhage pose significant challenges in clinical management. Despite advances in trauma care, comprehensive documentation of successful management strategies for severe open pelvic fractures with multiple complications remains limited.

**Patient concerns:**

A 36-year-old female presented with an open pelvic fracture following a motor vehicle collision, complicated by hemorrhagic shock (blood pressure 66/45 mm Hg), multiple organ injuries including bladder rupture and rectal damage, and an 18-cm open wound from the right groin to the anus with active bleeding.

**Diagnoses:**

Young and Burgess anterior-posterior compression III type pelvic fracture with vertical shear mechanism, bilateral internal iliac artery injuries, complete bladder and urethral rupture, and extensive perineal-rectal injuries.

**Interventions:**

The patient underwent multiple staged interventions including emergency arterial embolization and stenting, external pelvic fixation, bladder repair, sigmoid colostomy, serial wound debridements, negative pressure wound therapy, and final reconstruction with muscle flap transposition and skin grafting. Treatment required 148 days of hospitalization, including 22 days in intensive care unit.

**Outcomes:**

The patient achieved successful recovery with healed fractures and wounds, though requiring permanent colostomy and cystostomy. Rehabilitation enabled functional recovery and return to daily activities.

**Lessons:**

Successful management of complex open pelvic fractures requires prompt hemorrhage control through interventional procedures, implementation of damage control principles with staged surgical approaches, close multidisciplinary collaboration, and comprehensive wound care and infection management. This strategy can significantly improve survival outcomes in severe cases.

## 1. Introduction

Open pelvic fractures constitute severe traumatic injuries typically resulting from high-energy impacts including motor vehicle collisions or falls from significant heights.^[1]^ These injuries frequently manifest with extensive hemorrhage, intra-abdominal organ damage, and urogenital system trauma, with mortality rates reaching 50%.^[2,3]^ Although recent advances in trauma care and multidisciplinary approaches have improved treatment outcomes for open pelvic fractures, these injuries continue to pose significant challenges in trauma management.^[4]^

Effective treatment requires prompt and efficient hemostasis, pelvic stabilization, and management of associated injuries.^[5]^ For severe open pelvic fractures, traditional single-stage surgical repair is often impractical and may exacerbate patient condition.^[6]^ Consequently, damage control concepts and staged surgical strategies have gained prominence in both research and clinical practice.^[2]^

We report the successful management of a complex open pelvic fracture with multiple organ injuries, demonstrating the effectiveness of damage control principles and coordinated multidisciplinary care in achieving favorable outcomes.

## 2. Case report

### 2.1. Current medical history

A 36-year-old female patient was transported by emergency medical services to the Emergency Department of Jiangnan University Affiliated Hospital in November 2023, presenting with “pelvic hemorrhage and altered consciousness for 30 minutes following truck compression injury.” The patient sustained truck-related compression trauma to the pelvic region approximately 30 minutes prior to hospital admission.

Post-trauma examination revealed significant ecchymosis and swelling in the right lower abdomen and hip region, with markedly limited lower extremity mobility. An 18-cm contaminated open wound extending from the right inguinal area to the anus was observed, demonstrating active hemorrhage with exudation of contaminants. Clinical findings included external rotation of the right lower limb, cool and clammy skin, and impaired consciousness.

### 2.2. Past history

The patient maintained good health status with no documented chronic medical conditions. No previous surgical interventions or significant trauma were reported.

### 2.3. Physical examination

Upon emergency department admission, the patient presented with the following vital parameters: temperature 36.5°C, heart rate 157 beats/min, respiratory rate 36 breaths/min, and blood pressure 66/45 mm Hg. The patient exhibited somnolence with cold and clammy skin. Physical assessment revealed positive pelvic compression-distraction test with notable crepitus. Significant ecchymosis and edema were observed in the right lower quadrant and ipsilateral hip region.

**Figure 1.**
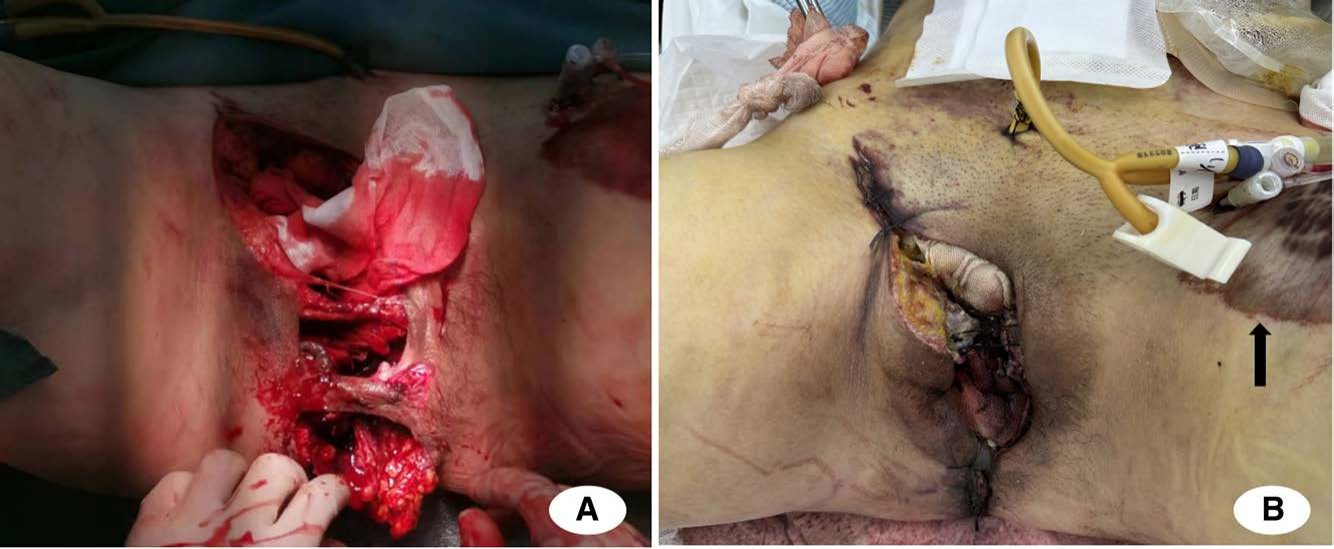
(A) Deep laceration from groin to anus. (B) Partial skin necrosis visible in the left hip area after the first surgery (black arrow).

An 18-centimeter contaminated laceration extended from the right inguinal area to the perianal region (Fig. 1A), demonstrating active hemorrhage with exudative discharge. The right lower extremity displayed external rotation deformity accompanied by substantial swelling. Point-of-care ultrasonography of the pelvic cavity demonstrated hypoechoic regions, suggestive of fluid accumulation.

### 2.4. Ancillary examinations

The patient underwent comprehensive laboratory evaluation upon admission. Blood analysis revealed hemoglobin level of 122 g/L, red blood cell count of 3.97 × 10^12^/L, and d-dimer concentration of 5.18 mg/L. Whole-body computed tomography examination demonstrated multiple fractures involving S1–S5 vertebrae and partial coccygeal bones. Bilateral fractures of the superior and inferior pubic rami were noted, accompanied by pubic symphysis diastasis.

**Figure 2.**
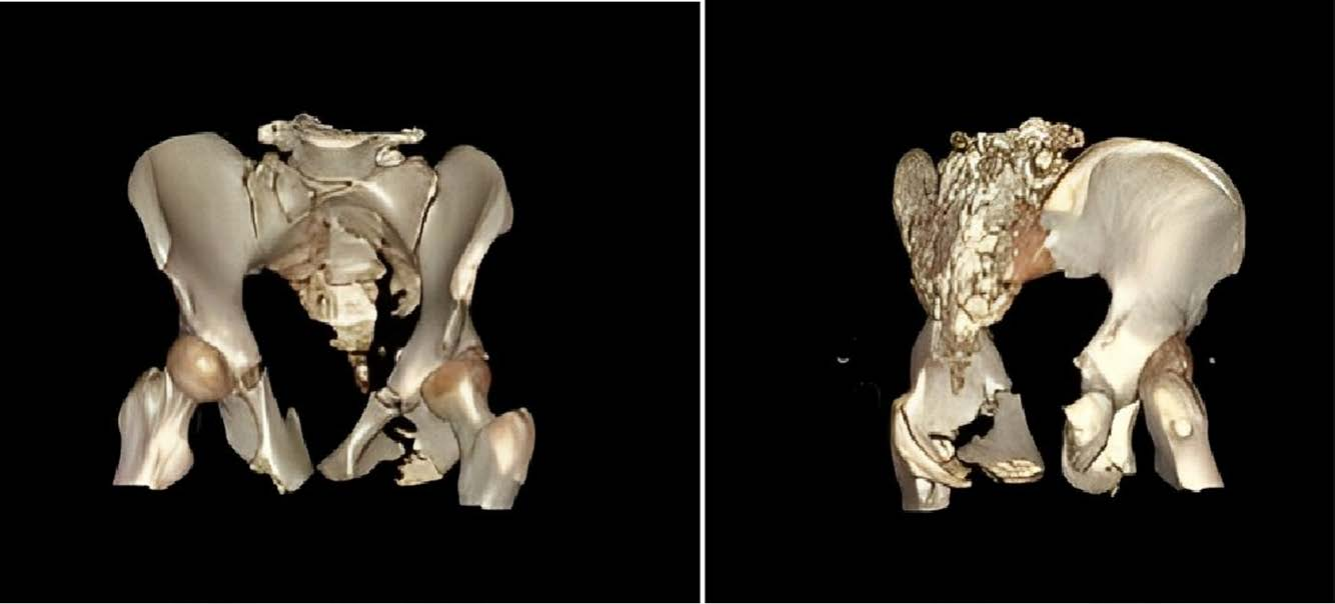
3D reconstruction showing comminuted pelvic fracture with displacement.

**Figure 3.**
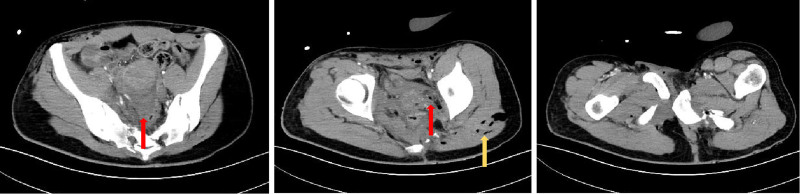
Computed tomography (CT) of the pelvis demonstrating an APC III type fracture, with fluid accumulation and free gas (red arrows), and extensive soft tissue damage (yellow arrows). APC = anterior-posterior compression .

Pelvic hematoma was present with associated muscular and soft tissue injuries (Figs. 2 and 3). The bladder wall appeared indistinct, suggesting possible injury. Minor pelvic pneumatosis was detected. The middle and distal segments of the right internal iliac artery showed evidence of injury with local extravasation. Additionally, the uterine cavity demonstrated mild distention with fluid accumulation.

### 2.5. Admission diagnosis

Young and Burgess type anterior-posterior compression-III pelvic fracture with vertical shear mechanism (VS type), complex pelvic ring disruption with extensive soft tissue injuries, urinary bladder rupture, complete urethral disruption, anal canal and rectal injuries, perineal soft tissue trauma, right internal iliac artery injury, and traumatic hemorrhagic shock.

### 2.6. Treatment process and perioperative care management

**Figure 4.**
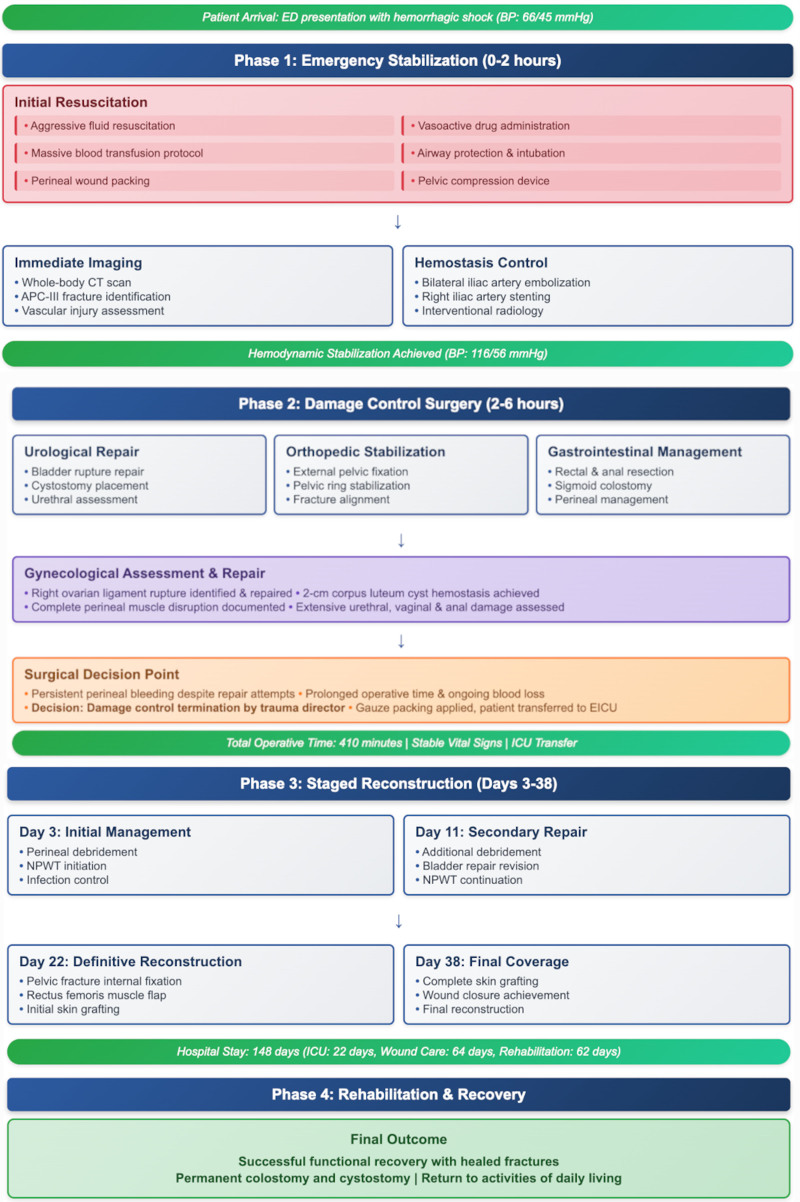
Treatment algorithm for severe open pelvic fracture: 4-phase management approach.

**Figure 5.**
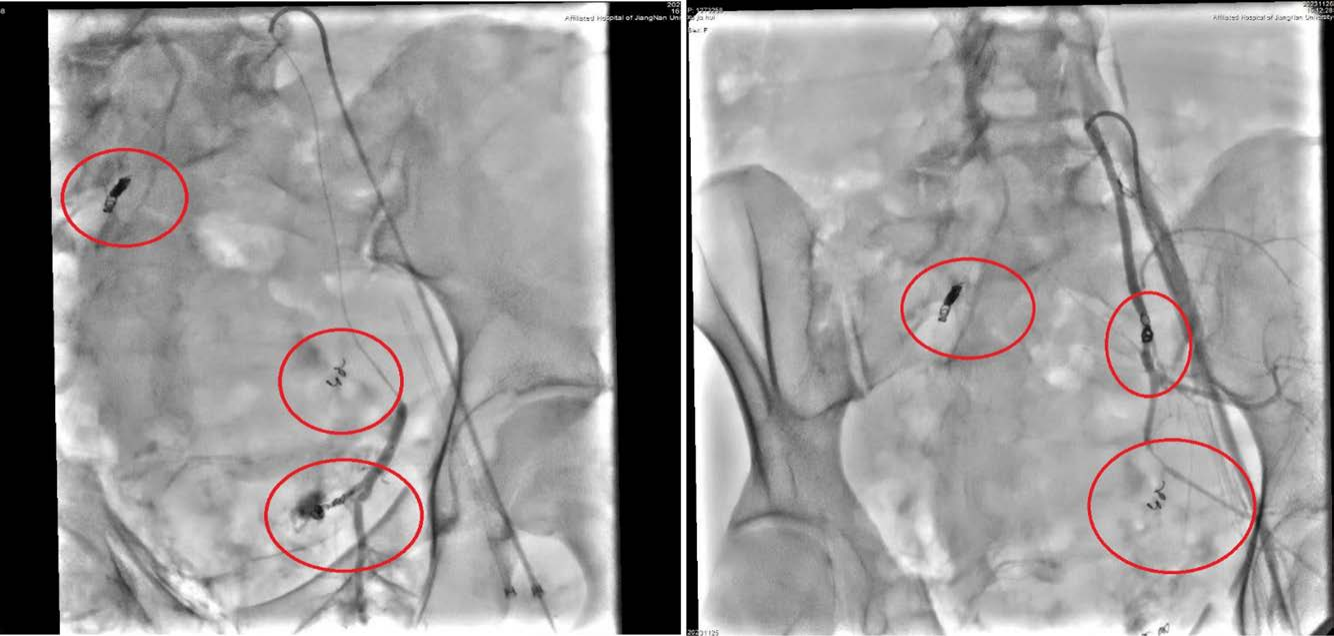
Selective embolization of the left internal iliac artery (2 red circles) and covered stent implantation in the right iliac artery (1 red circle).

The patient’s management followed a systematic 4-phase approach based on damage control principles (Fig. 4). Phase 1 (emergency stabilization, 0–2 hours) focused on immediate lifesaving interventions including aggressive fluid resuscitation, massive transfusion protocol activation, airway protection with endotracheal intubation, and hemodynamic stabilization. Concurrent perineal wound packing and pelvic compression device application provided temporary hemorrhage control. Whole-body computed tomography imaging identified the extent of injuries, followed by urgent interventional radiology procedures including bilateral internal iliac artery embolization and right iliac artery stent placement, successfully achieving hemodynamic stability (Fig. 5).

Phase 2 (damage control surgery, 2–6 hours) encompassed multidisciplinary surgical interventions addressing multiple organ systems. Urological repair included bladder rupture closure and cystostomy placement. Orthopedic stabilization involved external pelvic fixation for fracture alignment. Gastrointestinal management required rectal and anal canal resection with sigmoid colostomy creation. Gynecological assessment revealed right ovarian ligament rupture and corpus luteum cyst bleeding, which were surgically addressed. However, persistent perineal bleeding and extensive tissue damage necessitated damage control termination by the trauma director, with gauze packing applied before emergency intensive care unit transfer.

**Figure 6.**
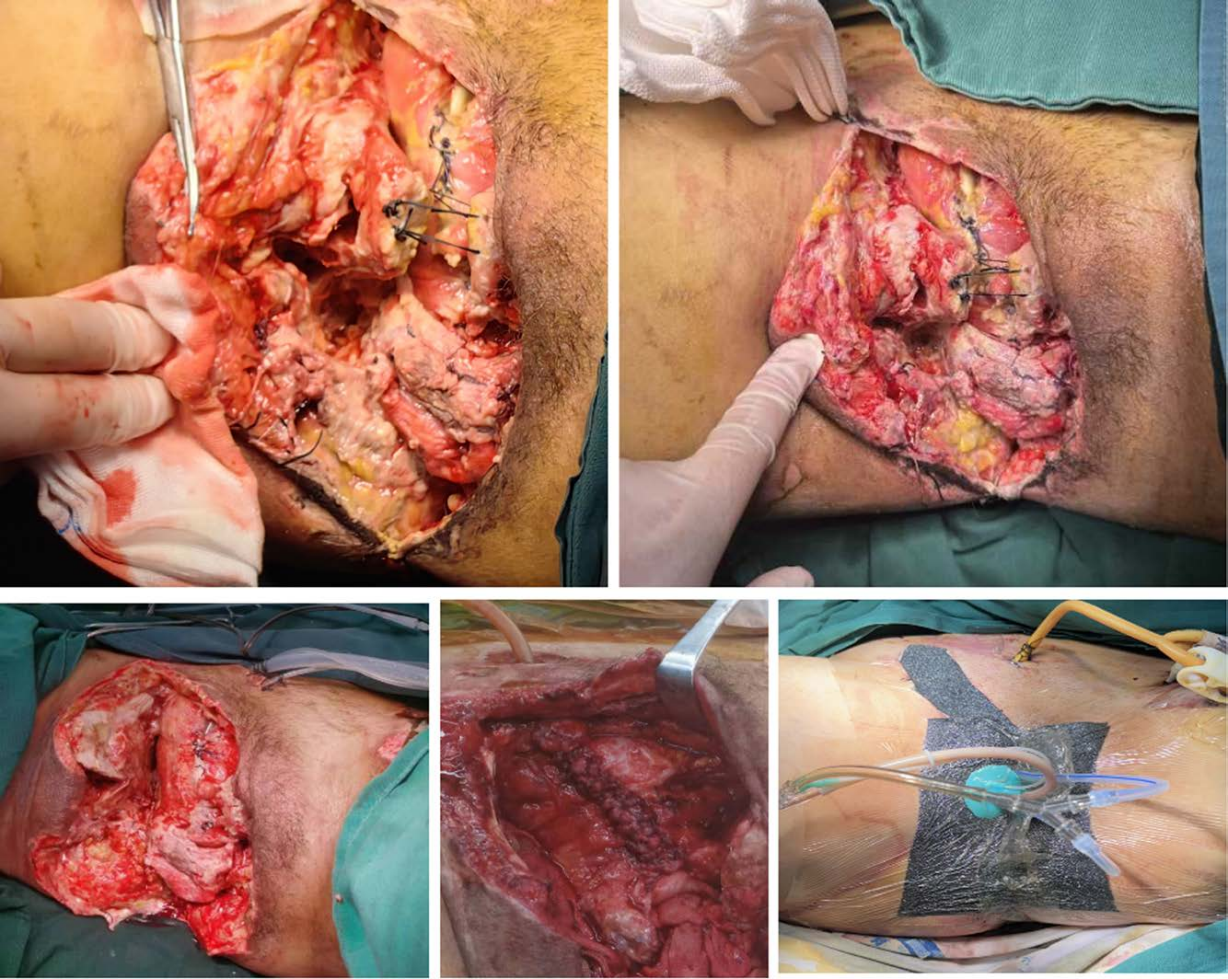
Sequential images illustrate multiple perineal debridements and repeated bladder repair surgeries due to recurrent urinary leakage, alongside the application of negative pressure wound therapy for perineal wound management.

**Figure 7.**
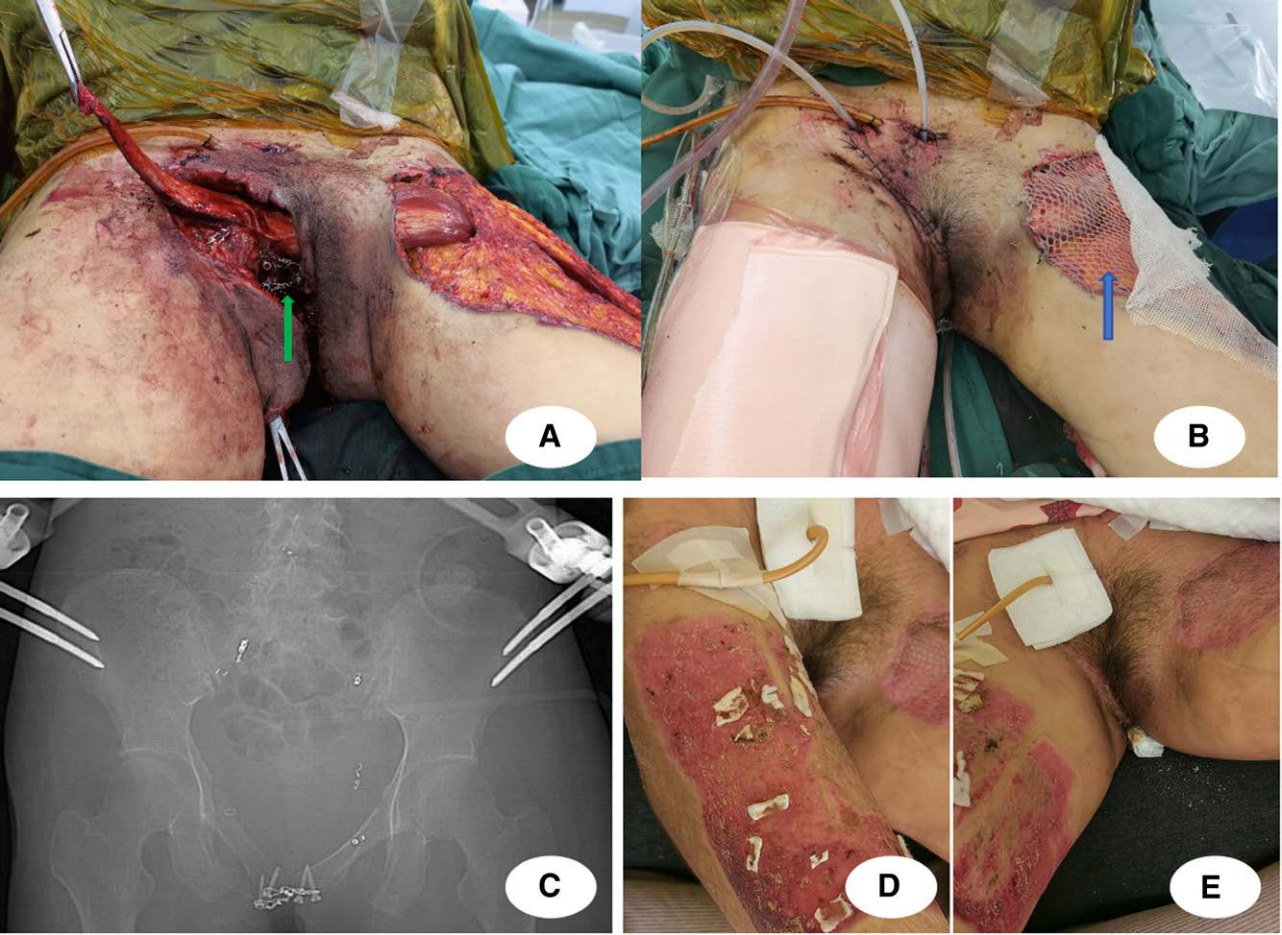
(A) Postoperative radiographs demonstrating bone stabilization. (B) Reconstruction and internal fixation of the pubic symphysis, with transposition of the left rectus femoris muscle flap (red arrow). (C) Wound closure achieved through split-thickness skin grafting (blue arrow). (D) and (E) Demonstrate successful wound healing.

Phase 3 (staged reconstruction, days 3–38) implemented planned sequential procedures once hemodynamic stability was maintained. Initial interventions included serial debridements and negative pressure wound therapy (NPWT) initiation. Subsequent procedures involved bladder repair revisions, pelvic fracture internal fixation, rectus femoris muscle flap transposition, and progressive skin grafting to achieve definitive wound closure (Figs. 6 and 7).

**Figure 8.**
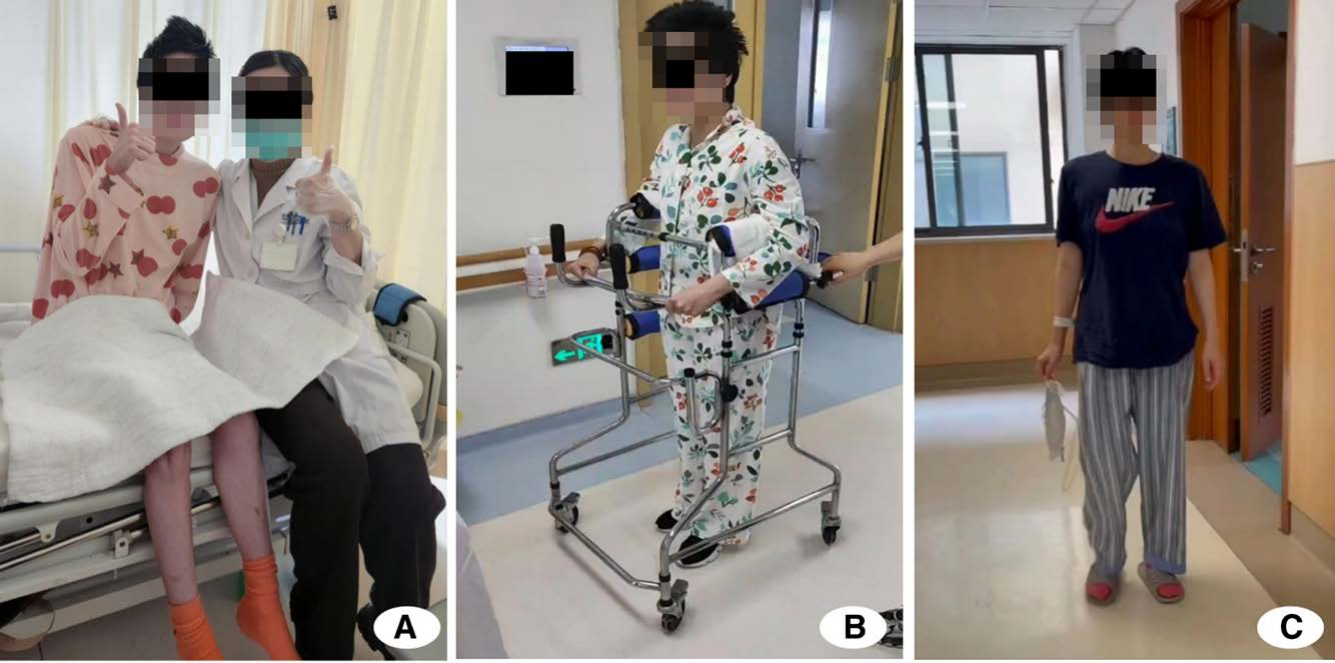
(A) The initial transition from supine to sitting position was observed 115 d post-injury, (B) assisted walking training was implemented 140 d post-injury, and (C) independent ambulation was attained 178 d post-injury.

Phase 4 (rehabilitation and recovery) after 148 days of hospitalization, the patient achieved complete wound healing and solid fracture union. During discharge examination, perineal wounds showed excellent healing with healthy granulation tissue and no signs of infection (Fig. 7D–E). Postoperative radiographs confirmed satisfactory pelvic fixation with proper hardware alignment (Fig. 8A). Progressive mobilization training was initiated using assistive devices, advancing from wheelchair to walker-assisted ambulation (Fig. 8B; see Video, Supplemental Digital Content 1). At 12-month follow-up, the patient demonstrated independent walking ability and resumed normal daily activities (Fig. 8C). Despite requiring permanent ostomies, functional recovery exceeded expectations with successful community reintegration.

Perioperative nursing management: Advanced perioperative care protocols proved essential for optimal patient outcomes. Critical care nursing encompassed invasive hemodynamic monitoring through arterial and central venous access, coupled with 4-day mechanical ventilation management utilizing evidence-based weaning strategies. Early nutritional intervention via jejunostomy tube placement on postoperative day 5 prevented protein-energy malnutrition while supporting tissue repair. Systematic infection surveillance incorporated daily wound assessments and microbiological monitoring, enabling prompt antimicrobial adjustments. Multimodal pain management protocols addressed both acute surgical pain and chronic discomfort associated with prolonged immobilization. Recognition and treatment of acute stress disorder through psychological counseling initiated on day 5 demonstrated comprehensive attention to mental health aspects of trauma recovery. Specialized wound care teams managed complex NPWT systems and serial dressing changes, maintaining optimal healing environments throughout the 22-day intensive care period. This integrated approach, extending beyond traditional physiological monitoring to encompass nutritional optimization, psychological support, and advanced wound management, exemplifies contemporary trauma nursing excellence and directly contributed to successful functional recovery.

Initially, *imipenem* was administered for infection control. On the seventh day of hospitalization, blood cultures identified *Acinetobacter baumannii*, while cultures of drainage fluid revealed both *Pseudomonas aeruginosa* and *A baumannii*. Based on antimicrobial susceptibility testing results, the antibiotic regimen was adjusted: *Imipenem* was discontinued, and combination therapy with vancomycin (1 g intravenously every 8 hours) and *polymyxin B* was initiated. Subsequent clinical and laboratory indicators demonstrated progressive reduction in infection parameters.

## 3. Discussion

Open pelvic fractures with extensive perineal involvement represent among the most challenging injuries in trauma surgery, with mortality rates historically approaching 50% but recently reduced to 23% through improved management protocols.^[4,7,8]^ This case report demonstrates successful management of a Young-Burgess anterior-posterior compression-III pelvic fracture complicated by bilateral vascular injuries, complete urogenital disruption, and extensive perineal trauma requiring multidisciplinary staged reconstruction.

### 3.1. Contemporary approaches to hemorrhage control in pelvic trauma

The retroperitoneal hemorrhage associated with severe pelvic fractures represents a unique clinical challenge due to the complex 3-dimensional anatomy of the pelvis and the extensive vascular networks involved.^[9]^ Unlike appendicular skeleton fractures, pelvic hemorrhage often originates from multiple sources: disrupted cancellous bone surfaces, torn muscular attachments, and injured branches of the internal iliac arterial system.^[10]^

Recent multicenter studies have demonstrated that the implementation of standardized hemorrhage control protocols, including early angioembolization within 60 minutes of presentation, reduces mortality from 42% to 19% in unstable pelvic fractures.^[11]^ The WSES guidelines now recommend determining intervention strategies for unstable pelvic fractures based on the patient’s hemodynamic status and FAST results: for patients with negative FAST but hemodynamic instability, urgent angiography and embolization should be considered; whereas patients with positive FAST and instability should undergo immediate laparotomy combined with preperitoneal pelvic packing or pelvic C-clamp application.^[12]^

In the present case, the complexity of bilateral internal iliac arterial injuries necessitated a hybrid approach combining selective coil embolization with covered stent grafting – a technique that has gained prominence in recent literature for managing complex pelvic vascular trauma.^[13]^ This approach allows for precise hemorrhage control while maintaining collateral circulation, particularly important in young patients where preservation of pelvic perfusion may impact long-term sexual and reproductive function.^[14]^

The decision to involve vascular surgery expertise reflects the growing recognition that complex pelvic trauma often requires subspecialty input beyond traditional trauma surgery. Recent institutional reviews suggest that early vascular surgery consultation in cases with persistent bleeding after initial embolization attempts reduces both transfusion requirements and overall mortality.^[15]^

### 3.2. Damage control principles and staged surgical management

The damage control paradigm prioritizes immediate life-threatening issues over anatomical restoration, proving essential in complex pelvic trauma where simultaneous hemorrhage, contamination, and organ injury can exceed physiologic tolerance.^[16]^ The 3-phase strategy – initial damage control, physiologic optimization, and definitive reconstruction – enables systematic management of competing priorities while minimizing operative burden.^[17]^

Our patient exemplified this approach through sequential interventions: emergent hemorrhage control and contamination management, followed by serial debridements with negative pressure therapy, culminating in delayed reconstruction at 22 days. This timeline reflected deliberate assessment of physiologic reserve, infection eradication, and optimal wound conditions-critical determinants of reconstructive success. The staged approach prevented physiologic exhaustion while systematically addressing each injury component, ultimately achieving functional restoration despite extensive initial trauma.

### 3.3. Multidisciplinary collaboration and nursing management

Successful management of complex pelvic trauma requires seamless integration of multiple specialties including trauma surgery, interventional radiology, orthopedics, urology, plastic surgery, and critical care.^[18]^ Evidence suggests that institutions with formal multidisciplinary trauma protocols demonstrate improved outcomes and reduced complications compared to traditional single-specialty management approaches.^[5]^

Perioperative nursing management represents a critical yet often underemphasized component of care. Key nursing interventions include hemodynamic monitoring, ventilator weaning protocols, infection surveillance, nutritional assessment, and psychological support.^[4]^ Our experience confirms that specialized wound care nursing significantly impacts healing outcomes in complex reconstructive cases, particularly regarding NPWT management and early recognition of complications.^[19]^

The psychological impact of severe pelvic trauma, particularly in young female patients with permanent functional alterations, requires specialized attention. Early psychological intervention, as implemented in this case, has been associated with improved long-term quality of life and reduced incidence of post-traumatic stress disorder.^[20,21]^

### 3.4. Comparison with similar cases and treatment advantages

Our case demonstrates several management advantages that likely contributed to the favorable outcome: rapid vascular intervention preventing exsanguination,^[22]^ appropriate application of damage control principles with staged reconstruction,^[23]^ comprehensive infection prevention and antimicrobial stewardship,^[24]^ and coordinated multidisciplinary care with specialized nursing support.^[23]^

Contemporary case series support our therapeutic strategy for severe pelvic trauma management. Freigang et al^[24]^ achieved 3.2% infection rates utilizing staged reconstruction protocols in high-energy fractures, while Kanakaris et al^[25]^ demonstrated 93% infection eradication through early vascular intervention despite mean ISS scores of 27.7. Our case validates these established principles, confirming that immediate hemorrhage control combined with damage control surgery significantly improves outcomes in complex pelvic injuries with extensive contamination and multiple organ involvement.

## 4. Conclusion

Complex open pelvic fractures require sophisticated multidisciplinary management integrating damage control principles with staged reconstruction protocols. Emergent angioembolization remains the cornerstone of hemorrhage control in hemodynamically unstable patients, though procedural complexity necessitates careful risk-benefit assessment and timely intervention modification when clinically indicated. Successful outcomes depend upon seamless interprofessional coordination among trauma surgery, interventional radiology, orthopedics, and critical care teams. Adjunctive therapies, including early nutritional optimization and targeted antimicrobial stewardship, significantly influence long-term functional recovery. This case validates the efficacy of systematic damage control management in severe pelvic trauma and provides evidence-based guidance for similar complex presentations requiring extensive reconstruction.

## Acknowledgments

We extend our gratitude to the multidisciplinary team at our hospital for their collaborative efforts. We also appreciate the understanding and cooperation of the patient and their family.

## Author contributions

**Conceptualization:** Haixiang Ding, Jianhong Zhou, Jingshi Pan.

**Resources:** Mingyang Yuan.

**Visualization:** Wenwen Wang, Mingyang Yuan, Junjan Tang.

**Writing** – **original draft:** Haixiang Ding.

**Writing** – **review & editing:** Haixiang Ding, Jingshi Pan.
